# Supplementary material for: Cranial osteology, taxonomic reassessment, and phylogenetic relationships of the Early Cretaceous (Aptian-Albian) turtle Trinitichelys hiatti (Paracryptodira)
Source: PeerJ. 2022 Nov 2;10:e14138. doi: 10.7717/peerj.14138 (PMC9636874; doi:10.7717/peerj.14138)
Supplement: Supplemental Information 1 [file peerj-10-14138-s001.docx]

**Supplementary information**

**Scorings changed from the matrix of Rollot et al. (2021)**

*Arundelemys* *dardeni*, *Lakotemys* *australodakotensis*, and now *Trinitichelys* *hiatti* have benefited from redescriptions over the last year. *A. dardeni* and *T. hiatti* were present in the last version of the matrix, while *L. australodakotensis* was newly added herein. As one of the goals of the present study is to reinvestigate the phylogenetic relationships of basal baenids, we reviewed the scorings for *A. dardeni* and *T. hiatti* and highlight the changes made below. We additionally updated the scorings for *Pleurosternon bullockii* following latest insights about variability within the shell of the latter (Guerrero & Perez-Garcia, 2020, 2021) as well as the scorings of *Eileanchelys waldmani* for characters 28, 57, 96, 97, 98, and 99, *Compsemys victa* for character 27, and *Kallokibotion bajazidi* for character 96 as 3D models were available to us for the latter three taxa. The scorings of *Peckemys brinkman* for characters 46 and 47, *Helochelydra nopcsai* for character 96, and *Naomichelys speciosa* for character 96 were also updated as we noticed mistakes in the latter.

**Character 1, skull shape in dorsal view:** changed for *Trinitichelys hiatti* (0->1).

**Character 8, palatine contribution to triturating surface:** changed for *Trinitichelys hiatti* (0->1).

**Character 13, prefrontal exposure on skull roof:** changed for *Trinitichelys hiatti* (1->0).

**Character 18, ventral extension of the jugal:** changed for *Trinitichelys hiatti* (1->0).

**Character 20, squamosal-parietal contact:** changed for *Trinitichelys hiatti* (1->0) and *Arundelemys dardeni* (1->?).

**Character 21, posteromedial margin of parietals:** changed for *Arundelemys dardeni* (1->?).

**Character 22, posterior thinning of parietals towards temporal margins:** changed for *Arundelemys dardeni* (0->?).

**Character 23, praepalatine foramen:** changed for *Trinitichelys hiatti* (1->0).

**Character 25, midline contact of pterygoid:** changed for *Trinitichelys hiatti* (?->2) and *Arundelemys dardeni* (1->2).

**Character 27, epipterygoid:** changed for *Compsemys victa* (0->?).

**Character 28, contribution of opisthotic to stapedial foramen:** changed in *Eileanchelys waldmani* (?->0), *Trinitichelys* *hiatti* (1->0), and *Arundelemys* *dardeni* (1-> 0&1).

**Character 35, preneural:** changed for *Pleurosternon bullockii* (0->0/1) following study of Guerrero & Perez-Garcia (2021).

**Character 39, vertebral length versus width:** changed for *Trinitichelys hiatti* (1->0).

**Character 46, gulars:** changed for *Pleurosternon bullockii* (1->0/1) following study of Guerrero & Perez-Garcia (2021) and *Peckemys brinkman* (1->0).

**Character 47, medial contact of extragulars:** changed for *Peckemys brinkman* (0->1).

**Character 57, horizontal tubercles on basioccipital:** changed for *Eileanchelys waldmani* (0->1).

**Character 58, length between orbit and cheek emargination:** changed for *Arundelemys dardeni* (?-> 0).

**Character 61, supraoccipital exposure on skull roof:** changed for *Trinitichelys hiatti* (0->1) and *Arundelemys dardeni* (0->?).

**Character 63, parietal width versus length:** changed for *Trinitichelys hiatti* (0->1) and *Arundelemys dardeni* (0->?).

**Character 65, cheek emargination:** changed for *Arundelemys dardeni* (0->1).

**Character 72, size of tympanum:** changed for *Arundelemys dardeni* (?->0).

**Character 78, shell sculpturing:** changed for *Arundelemys dardeni* (1->?).

**Character 93, entoplastral dimensions:** changed for *Pleurosternon bullockii* (2->1/2) according to Guerrero & Perez-Garcia (2020, 2021).

**Character 96, basipterygoid process:** changed for *Eileanchelys waldmani* (?->1), *Helochelydra nopcsai* (1->?), *Naomichelys speciosa* (1->?), and *Kallokibotion bajazidi* (2->1).

**Character 97, anterior tubercula:** changed for *Eileanchelys waldmani* (?->0).

**Character 98, prootic in trigeminal foramen:** changed for *Eileanchelys waldmani* (?->0).

**Character 99, enclosure of internal carotid artery in bone:** changed for *Eileanchelys waldmani* (?->0).

**Characters changed**

**Character 9, interorbital width:** (0) wide (70–100% of skull width), (1) moderate (35–65% of skull width), (2) narrow (0–30% of skull width).

*Previous definition:* interorbital width: (0) wide, (1) narrow (hour-glass-shaped between the orbits).

*Comments:* We changed the definition of this character as a great range of widths can be observed in turtles, ranging from the expanded interorbital width of *Proganochelys quenstedti* (Gaffney, 1990; Supp. Fig. 1A), in which the orbits are fully vertical and the interorbital space actually corresponds to the entire width of the skull at that level, to the intermediate width of *Pleurosternon bullockii* (Evers, Rollot & Joyce, 2020; Supp. Fig. 1B), and the extremely narrow interorbital width of *Sandownia harrisi* (Evers & Joyce, 2020; Supp. Fig. 1A). The interorbital width was estimated in dorsal view by measuring the narrowest width between the orbits on the skull roof relative to the width of the skull at that level. Character state 0 corresponds to an interorbital space that occupies 70–100% of the skull width, character state 1 corresponds to an interorbital space that occupies 35–65% of the skull width, and character state 2 corresponds to an interorbital space that occupies 0–30% of the skull width. Interorbital width values that fall between the percentages defined for the character states should be scored as polymorphic. As the extent of the interorbital width is linked with the orientation of the orbits (taxa with dorsally oriented orbits tend to have reduced interorbital widths), we removed character 5 of the matrix of Rollot, Evers & Joyce (2021). The character is ordered.


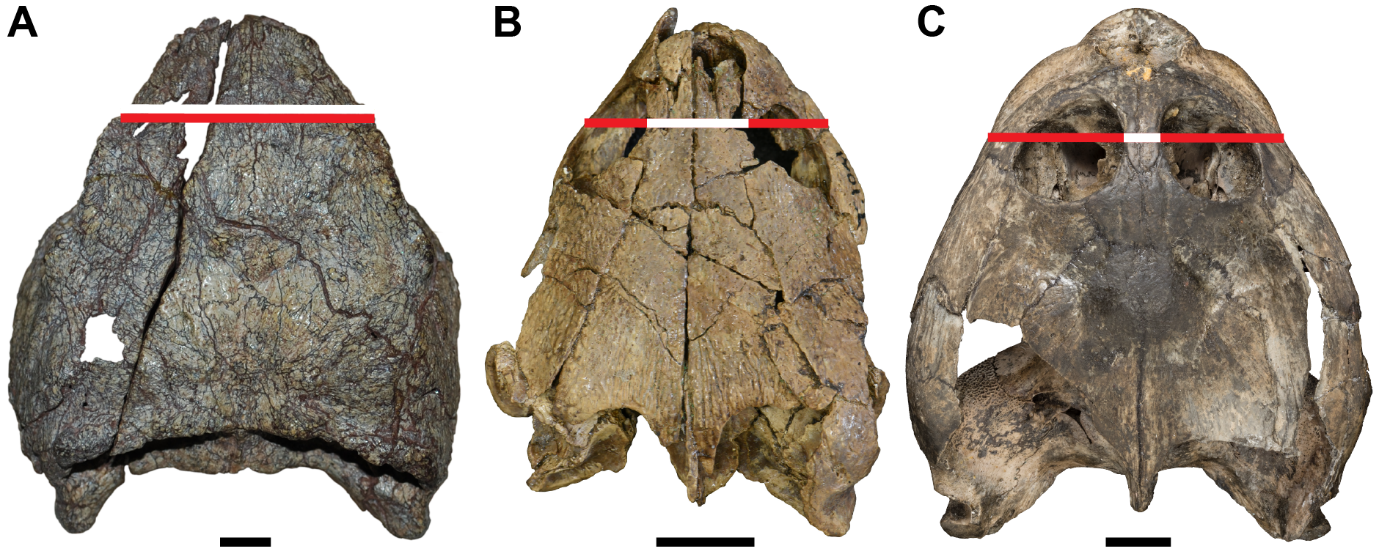


**Supplementary Figure 1. Illustration of character 9.** (A), state 0 (*Proganochelys quenstedti*, SMNS 16980). (B), state 1 (*Pleurosternon bullockii*, UMZC T1041). (C) state 2 (*Sandownia harrisi*, MIWG 3480). White bars indicate interorbital width and red bars indicate skull width at the same level. Scale bar equals 10 mm.

**Character 11, nasal size:** (0) large, more than 10% of surface of the interorbital skull roof, (1) small to absent, less than 10% of surface of the interorbital skull roof.

*Previous definition:* nasal size: (0) large, (1) reduced or absent.

*Comments:* We noticed that estimating the nasal size and distinguishing a large nasal from a small one within paracryptodires might be somewhat difficult and that a boundary between large and small is not always easy to determine visually. We, therefore, measured the surface of the nasal in dorsal view relative to the skull roof surface taken between the anterior tip of the nasals and an imaginary line drawn between the posteriormost limit of the orbits (Supp. Fig. 2). The nasal is considered large if the ratio is greater than 10% and small if the ratio is below 10%. The surfaces were measured on the combined left and right sides of the skull whenever it was possible (Supp. Figs. 2B & 2C). If one side is missing or too much damage or shearing affects one side of the skull, the measurements then should only be taken on the side of the skull that fully preserves the respective nasal and interorbital space (Supp. Figs. 2E & 2F).


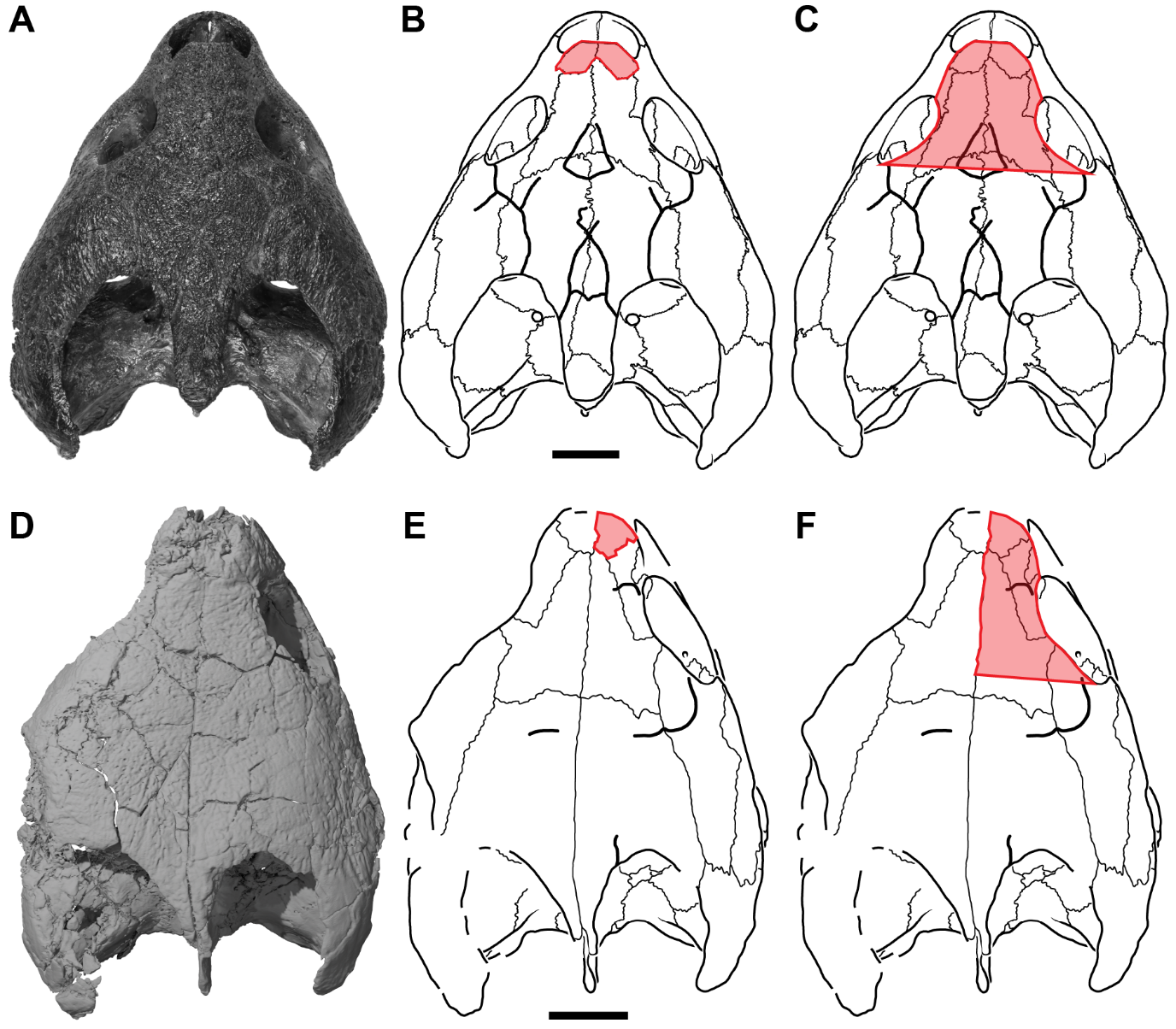


**Supplementary Figure 2. Illustration of surface measurements for character 11.** (A), dorsal view of *Eubaena cephalica* (DMNH 96004). (B), state 0 with measurements of the nasal taken on the combined left and right sides of *Eubaena cephalica* (DMNH 96004; following drawings of Rollot, Jyson & Joyce, 2018). (C), state 0 with measurements of the interorbital surface taken on the combined left and right sides of *Eubaena cephalica* (DMNH 96004; followings drawings of Rollot, Lyson & Joyce, 2018). (D), dorsal view of *Trinitichelys hiatti* (MCZ VPRA-4070. (E), state 1 with measurements of the nasal taken only on the right side of the skull of *Trinitichelys hiatti* (MCZ VPRA-4070). (F), state 1 with measurements of the interorbital surface taken only on the right side of the skull of *Trinitichelys hiatti* (MCZ VPRA-4070). Scale bars equal 10 mm.

**Character 26, posterior process of the pterygoid:** (0) absent and prootic exposed ventrally, (1) short, prootic not exposed ventrally and no contact with the basioccipital, (2) moderately long with point contact with the basioccipital, (3) elongate and extensive contact with the basioccipital.

*Previous definition:* pterygoid-basioccipital contact: (0) absent, (1) present but poorly developed, posterior process of pterygoid short, (2) present and extensive, posterior process of pterygoid elongate.

*Comments:* Variation occurs within stem turtles in that the prootic might be exposed ventrally, as observed in *Eileanchelys waldmani* and *Chubutemys copelloi*, or might be covered by a short posterior pterygoid process that does not contact the basioccipital, as is the case in *Mongolochelys efremovi*. As character state (0) of the previous character definition did not allow perceiving this distinction, we modified the character to take this amount of variation into account. Character is run ordered.


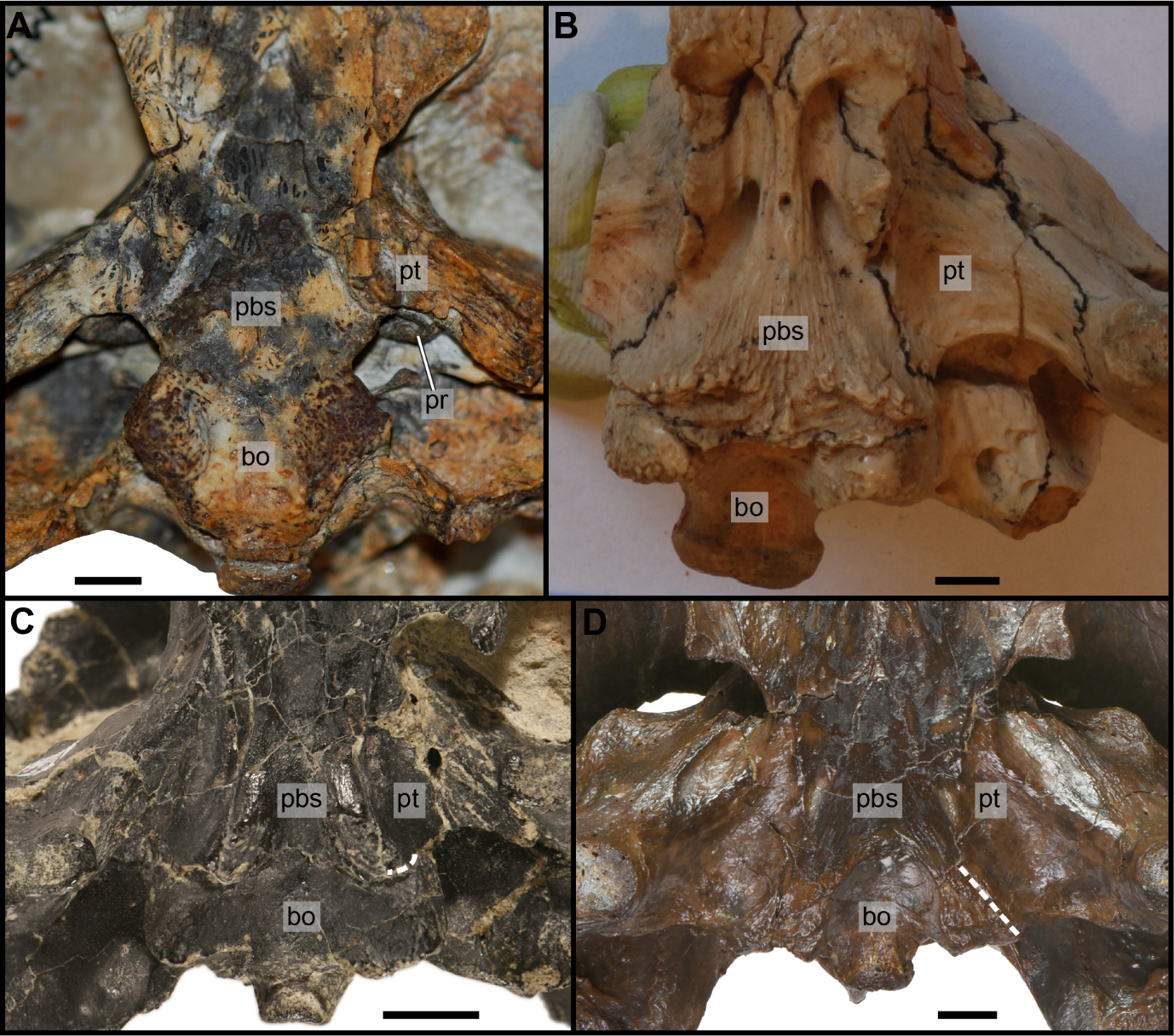


**Supplementary Figure 3. Illustration of character 26.** (A), state 0 (*Chubutemys copelloi*, MPEF–PV 1236; picture is courtesy of Márton Rabi). (B), state 1 (*Mongolochelys efremovi*, PIN 552–459; picture is courtesy of Márton Rabi). (C) state 2 (*Uluops uluops*, UCM 53971). (D) state 3 (*Eubaena cephalica*, DMNH 96004). White dashed lines indicate the pterygoid–basioccipital suture. Scale bar equals 5 mm. Abbreviations: **bo**, basioccipital; **pbs**, parabasisphenoid; **pr**, prootic; **pt**, pterygoid.

**Characters deleted**

**Character 5, orientation of orbit:** (0) laterally, (1) dorsally.

*Comment:* we choose to remove this character as it is now dependent on the new definition of character 9, interorbital width.

**Character 25: posterior margin of vomer:** (0) straight, (1) pointed posteriorly.

*Comment:* the observed differences in the dataset appear to be too subtle to score the character objectively.
